# A New Strategy for Detecting Plant Hormone Ethylene Using Oxide Semiconductor Chemiresistors: Exceptional Gas Selectivity and Response Tailored by Nanoscale Cr_2_O_3_ Catalytic Overlayer

**DOI:** 10.1002/advs.201903093

**Published:** 2020-02-24

**Authors:** Seong‐Yong Jeong, Young Kook Moon, Tae‐Hyung Kim, Sei‐Woong Park, Ki Beom Kim, Yun Chan Kang, Jong‐Heun Lee

**Affiliations:** ^1^ Department of Materials Science and Engineering Korea University Seoul 02841 Republic of Korea

**Keywords:** ethylene sensors, fruit ripening, metal oxide gas sensors, oxide semiconductor chemiresistors, plant hormones

## Abstract

A highly selective and sensitive detection of the plant hormone ethylene, particularly at low concentrations, is essential for controlling the growth, development, and senescence of plants, as well as for ripening of fruits. However, this remains challenging because of the non‐polarity and low reactivity of ethylene. Herein, a strategy for detecting ethylene at a sub‐ppm‐level is proposed by using oxide semiconductor chemiresistors with a nanoscale oxide catalytic overlayer. The SnO_2_ sensor coated with the nanoscale catalytic Cr_2_O_3_ overlayer exhibits rapid sensing kinetics, good stability, and an unprecedentedly high ethylene selectivity with exceptional gas response (*R*_a_/*R*_g_ − 1, where *R*_a_ represents the resistance in air and *R*_g_ represents the resistance in gas) of 16.8 at an ethylene concentration of 2.5 ppm at 350 °C. The sensing mechanism underlying the ultraselective and highly sensitive ethylene detection in the unique bilayer sensor is systematically investigated with regard to the location, configuration, and thickness of the catalytic Cr_2_O_3_ overlayer. The mechanism involves the effective catalytic oxidation of interfering gases into less‐ or non‐reactive species, without limiting the analyte gas transport. The sensor exhibits a promising potential for achieving a precise quantitative assessment of the ripening of five different fruits.

## Graphical abstract

A bilayer sensor with a SnO_2_ sensing layer and a catalytic Cr_2_O_3_ overlayer is proposed as a novel solution enabling the exclusive detection of sub‐ppm‐level ethylene, which is a representative plant hormone. The sensor can be applied in a wide range of applications that require the real‐time and onsite assessment of fruit freshness/ripening or the control of plant growth/development.

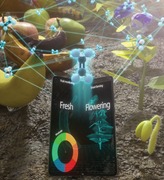

## 1 Introduction

**Figure 1:**
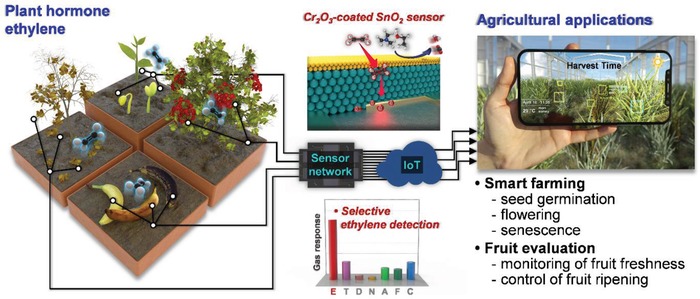
Concept and design of this study.

Ethylene (C_2_H_4_) is a representative gas hormone that stimulates the development and growth of plants in processes such as seed germination, flowering, leaf abscission, fruit ripening, and senescence^[^ 1, 2, 3, 4 ^]^; further, its physiological effects are observed even at extremely low concentrations (0.1–1 ppm) (**Figure** **1**, left).^[^ 2, 4, 5, 6 ^]^ Thus, a highly selective, sensitive, and cost‐effective detection of ethylene has considerable potential for numerous agricultural applications, and its impact has rapidly increased with the advancements in the Internet of Things and sensor networks (Figure 1, right). For instance, ethylene being the simplest alkene hydrocarbon, its detection has been used to evaluate the freshness of fruits and to facilitate the ripening of early‐harvested fruits.^[^ 7 ^]^ Some fruits, vegetables, and ornamental crops produce excessive amount of ethylene; therefore, uncontrolled ripening can cause a rapid deterioration of fresh product during storage and transportation.^[^ 3 ^]^ Accordingly, developing a method that can precisely measure and control sub‐ppm‐level of ethylene would provide new strategies for developing and storing plants and for assessing fruit freshness, ripening of fruits, and opening of flowers.

Various methods and instruments have been used to detect ethylene, including gas chromatography,^[^ 5 ^]^ photoluminescence quenching,^[^ 8 ^]^ fluorescent probes,^[^ 9, 10 ^]^ and photoacoustic spectrometers.^[^ 11 ^]^ However, the required equipment is bulky, expensive, and necessitates the use of time‐consuming gas sampling/pretreatment steps; these factors impede the accomplishment of an instantaneous, portable, and cost‐effective gas monitoring. Therefore, gas sensors using metal oxides,^[^ 12, 13, 14, 15, 16, 17, 18, 19 ^]^ carbon nanotubes (CNTs),^[^ 20, 21, 22 ^]^ and graphene‐based materials^[^ 23, 24, 25 ^]^ have been attracting attention as viable alternatives; this is associated with their high gas response, fast‐responding speed, simple sensor structure, facile miniaturization, and good stability. For the detection of ethylene with low reactivity, metal oxide semiconductors that operate at elevated temperatures (200–400 °C) are more advantageous than CNT‐ and graphene‐based chemiresistors, which are generally operated at room temperature or mildly heated conditions.

However, the simple sensing mechanism often provides similar chemiresistive variations for a wide range of gases; this mechanism is based on a surface reaction between the target gas and the ionized surface oxygen. Therefore, the highly selective and sensitive detection of ethylene has remained a challenge since the introduction of metal oxide semiconductor gas sensors in 1960s;^[^ 26 ^]^ this is despite the strong demand from the agricultural industry. It is considered that this problem is related to ethylene being relatively stable and thus less reactive than other highly reactive interfering gases (such as ethanol, formaldehyde, trimethylamine, dimethylamine, and ammonia); further, its high bond energy and non‐polarity are unfavorable for controlling the adsorption of a specific gas.^[^ 3, 27, 28 ^]^

Studies on ethylene sensing using oxide semiconductor chemiresistors are currently in the nascent stage. Although catalyst loading/doping has been employed to enhance the response to ethylene,^[^ 29, 30, 31 ^]^ this has frequently been accompanied by an increase in the responses to interfering gases and/or an increase in sensor resistance beyond the measurable range; consequently, this has hampered selective ethylene detection and/or resistance measurements, when using a conventional electric circuit. In addition, although sensor arrays consisting of multiple oxide‐based sensors^[^ 32 ^]^ have been investigated, their ability to discriminate and quantify sub‐ppm‐levels of ethylene is still insufficient for utilization in practical applications and for assessing fruit freshness.

Herein, we report a novel strategy for designing highly selective and sensitive ethylene gas sensors by using a SnO_2_ sensing film that is coated with a nanoscale Cr_2_O_3_ catalytic overlayer. The key concept of this intriguing bilayer sensor is the catalytic oxidation of highly reactive interfering gases into non‐ or less‐reactive species, such as CO_2_ and H_2_O, which was achieved without sacrificing the transport of the analyte gas to the lower sensing region and without affecting the sensor resistance. To demonstrate this concept and for securing a high ethylene response even after the selective catalytic filtering of gases, the micro‐thick films of SnO_2_ hollow spheres to exhibit high and comparable responses to a range of reducing gases have been used as sensing layers and Cr_2_O_3_ has been selected as the catalytic overlayer materials to reduce the effect of interference gas from the preliminary studies. The gas sensing characteristics of SnO_2_ sensing films with different configurations of the nanoscale Cr_2_O_3_ catalytic overlayer were studied and the potential of the proposed sensor for use in assessing fruit freshness is confirmed by investigating the ripening of five different fruit types (banana, apple mango, peach, kiwifruit, and blueberry). To the best of our knowledge, this paper presents the first report of a metal oxide gas sensor that enables the highly selective and sensitive detection of sub‐ppm‐levels of ethylene with negligible cross‐responses to other food‐related interfering gases (trimethylamine, dimethylamine, and ammonia), ubiquitous ethanol, and representative indoor pollutants (formaldehyde and carbon monoxide). The main aim of this study is to elucidate the sensing mechanism that underlies the exclusive detection of ethylene in relation to the sensing film, catalytic overlayer materials, and configuration of the bilayer films.

## 2 Results and Discussion

Sn‐containing precursor spheres were prepared using ultrasonic spray pyrolysis and converted into SnO_2_ spheres via heat treatment at 600 °C for 2 h (Figure S1a, Supporting Information). The average diameter of ≈100 spheres was 0.84 ± 0.36 µm. Hollow morphologies were observed in broken shells (inset in Figure S1a, Supporting Information), and these were further confirmed via transmission electron microscopy (TEM) analysis. Figure S1b, Supporting Information, shows a bright contour in the central region of the sphere, which is contrasting to the dark contour observed in the outer region. The shells exhibited thicknesses of ≈34 nm, and each sphere consisted of a number of primary particles with sizes of ≈15 nm (Figure S1c, Supporting Information). A high‐resolution TEM (HR‐TEM) image shows lattice patterns with interplanar spacings of 2.69 and 3.31 Å, corresponding to the (101) and (110) planes of SnO_2_ tetragonal structures, respectively (Figure S1d, Supporting Information). The N_2_ adsorption and desorption isotherms of the SnO_2_ hollow spheres exhibited type IV behavior with H3 hysteresis loops, indicating the presence of abundant mesopores (size: 2–50 nm) (Figure S2, Supporting Information). The Brunauer–Emmett–Teller (BET) surface area was 14.4 m^2^ g^−1^, which indicates that the SnO_2_ hollow spheres exhibited a highly gas‐accessible structure.^[^ 33 ^]^

**Figure 2:**
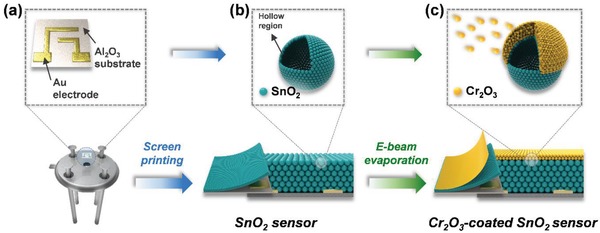
Process used to fabricate sensors: a) Al_2_O_3_ substrate with Au electrodes; b) formation of SnO_2_ sensing layer via screen printing; c) coating of Cr_2_O_3_ overlayer via e‐beam evaporation.

The sensing film was fabricated by screen printing a slurry containing SnO_2_ hollow spheres on an Al_2_O_3_ substrate with two Au electrodes, which was followed by heat treatment at 450 °C for 3 h (**Figure 2**a,b). Subsequently, a Cr_2_O_3_ catalytic overlayer was deposited on the SnO_2_ sensing film via electron‐beam (e‐beam) evaporation (Figure 2c). The thicknesses of the sensing film and catalytic overlayer were controlled by changing the emulsion thickness of the screen mask and the e‐beam evaporation time, respectively. Hereinafter, SnO_2_ sensors with 0.05‐, 0.3‐, and 0.6‐µm‐thick Cr_2_O_3_ overlayers are referred to as 0.05Cr_2_O_3_‐SnO_2_, 0.3Cr_2_O_3_‐SnO_2_, and 0.6Cr_2_O_3_‐SnO_2_, respectively.

**Figure 3:**
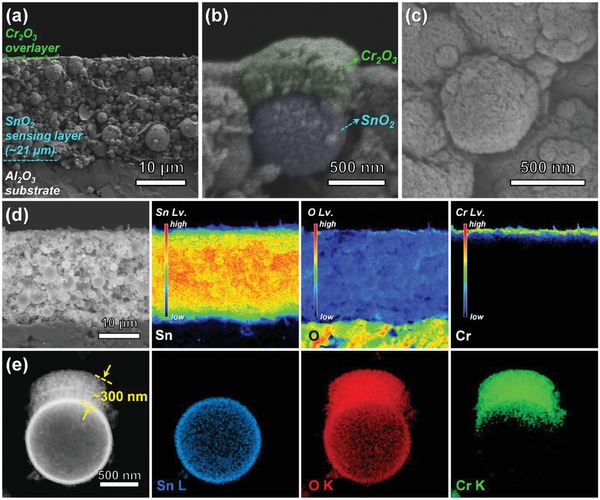
Microstructure and morphology characterization of the 0.3Cr_2_O_3_‐SnO_2_ sensor: a) cross‐sectional SEM image of the entire sensing film, b) high‐magnification SEM image of the uppermost region of the sensing film, c) top‐view image, d) backscattered image and EPMA elemental (Sn, O, and Cr) mapping, and e) backscattered image and TEM elemental (Sn, O, and Cr) mapping of a sphere detached from the uppermost region of the sensing film.

Cross‐sectional and top‐view scanning electron microscopy (SEM) images of the 0.3Cr_2_O_3_‐SnO_2_ bilayer sensor are shown in **Figure 3**a–c. The SnO_2_ sensing film has a thickness of ≈21 µm and uniform thickness (Figure 3a). A high‐magnification SEM image of the area near the top of the sensing film reveals that the spherical SnO_2_ powder is covered with Cr_2_O_3_ nanoparticles (Figure 3b). As shown in the top‐view image of the film in Figure 3c, the spheres have a rough surface morphology, whereas the SnO_2_ spheres in Figure S1a, Supporting Information, have a clean surface morphology, which verifies the presence of the coating of Cr_2_O_3_ nanoparticles on the SnO_2_ sensing film. Compositional variations of Sn, O, and Cr elements near the surface of the film were analyzed via electron probe microanalysis (EPMA) elemental mapping (Figure 3d). The majority of the Sn and O elements were found to be uniformly distributed throughout the entire sensing film, whereas the Cr element was located only in the uppermost region of the film. TEM analysis showed a 0.3‐µm‐thick Cr_2_O_3_ overlayer (Figure 3e) coated on the surface of SnO_2_ spheres, which were located in the upper part of the sensing film; this was consistent with the SEM and elemental mapping results (Figure 3b,d). The morphologies and microstructures of the 0.05Cr_2_O_3_‐SnO_2_ and 0.6Cr_2_O_3_‐SnO_2_ sensors were also analyzed (Figure S3, Supporting Information), and their thicknesses were both ≈22 µm (Figure S3a,f, Supporting Information), which were similar to that of the 0.3Cr_2_O_3_‐SnO_2_ sensor (≈21 µm). The SEM, EPMA, and TEM results clearly indicate the uniform coating of Cr_2_O_3_ layers with thicknesses of 0.05 and 0.6 µm on the thick SnO_2_ sensing film.

The phases and crystallinities of the pure SnO_2_ and 0.3Cr_2_O_3_‐SnO_2_ sensors were characterized using X‐ray diffraction (Figure S4, Supporting Information). The 0.3Cr_2_O_3_‐SnO_2_ specimen showed tetragonal SnO_2_ (ICDD #41‐1445) and rhombohedral Cr_2_O_3_ (ICDD #38‐1479) phases, and no second phase was found. The crystallite sizes of the polycrystalline SnO_2_ and Cr_2_O_3_ phases were calculated as 12.9 ± 0.5 and 19.5 ± 2.8 nm, respectively, by using the Scherrer's equation. No obvious peak shift was observed for the SnO_2_ peak of the 0.3Cr_2_O_3_‐SnO_2_ specimen, which indicates that Cr was not incorporated into the SnO_2_ lattice.

**Figure 4:**
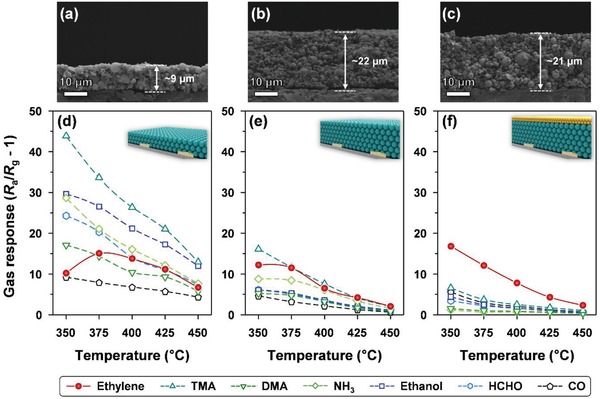
Cross‐sectional SEM images and gas‐sensing properties of the a,d) thin SnO_2_ sensor (thickness: ≈9 µm), b,e) thick SnO_2_ sensor (thickness: ≈22 µm), and c,f) 0.3Cr_2_O_3_‐SnO_2_ sensor (thickness: ≈21 µm) (concentration of the analyte gas: 2.5 ppm; temperature range: 350–450 °C).

**Figure** **4** shows the gas responses of two pure SnO_2_ sensors (with different film thicknesses of ≈9 and ≈22 µm) and the 0.3Cr_2_O_3_‐SnO_2_ sensor (SnO_2_ film thickness of ≈21 µm) to 2.5 ppm ethylene, trimethylamine (TMA), dimethylamine (DMA), ammonia (NH_3_), ethanol, formaldehyde (HCHO), and carbon monoxide (CO) at 350–450 °C. All three sensors (with and without the Cr_2_O_3_ overlayer) exhibited the typical chemiresistive variations of n‐type oxide semiconductor gas sensors; there were decreases and recoveries of sensor resistance upon exposure to reducing gas and air, respectively (Figure S5, Supporting Information). Accordingly, the gas response (S) was defined as follows: *R*_a_ */R*_g_ – 1.

For the thin SnO_2_ sensor with a film thickness of ≈9 µm (Figure 4a), the response to 2.5 ppm TMA was higher than those of the other gases over the entire range of sensing temperatures (350–450 °C), and the response to ethanol was the second‐highest (Figure 4d). The high responses to TMA and ethanol (see Figure 4d) were consistent with the typical gas‐sensing characteristics of pure SnO_2_ sensors reported in the literature.^[^ 33, 34, 35, 36, 37, 38 ^]^ However, because the responses to other gases were also substantially high, it was difficult to achieve a highly selective detection of a specific gas. When the thickness of the sensing film was increased from ≈9 to ≈22 µm (Figure 4b), the responses to the majority of the gases decreased (Figure 4e). We considered the plausible reasons for the overall decrease in gas responses, namely: 1) the limitation of the gas transport from the top to the lower sensing region close to the electrodes and 2) oxidation of the analyte gas into non‐reactive species (e.g., CO_2_ or H_2_O) in the upper part of the sensing film with catalytic activity. Both reasons are possible, considering the increase in the sensing‐film thickness and the fact that SnO_2_ is a good catalytic material for the oxidation of reducing gases.^[^ 39, 40 ^]^ However, it is worth noting that the increase in the thickness of the SnO_2_ sensor had barely any effect on the response to ethylene, whereas this change significantly decreased the responses to all the other gases (TMA, DMA, NH_3_, ethanol, HCHO, and CO). The negligible change in the ethylene response, despite the increase in film thickness, suggested that gas transport was not the critical limiting step for ethylene; thus, ethylene could diffuse to the lower part of the sensing film without significant oxidation. This explanation is plausible, considering that ethylene is a very stable gas; hence, its oxidation is relatively difficult.^[^ 3, 27, 30 ^]^ In contrast, the decrease in the gas responses to TMA, DMA, NH_3_, ethanol, HCHO, and CO could be attributed to the oxidative consumption of these gases in the thick SnO_2_ sensing film. However, as shown in Figure 4e, the responses to the seven different gases were not distinctively different from each other, and thus this method was considered to be insufficient for use in the selective detection of a specific gas.

However, the subsequent coating of a 0.3‐µm‐thick Cr_2_O_3_ overlayer on the SnO_2_ sensing film (Figure 4c) further reduced the responses to the majority of the gases, except for ethylene (Figure 4f). The 0.3Cr_2_O_3_‐SnO_2_ sensor invariably exhibited a remarkably high gas response to ethylene at 350 °C (*S* = 16.8 at 2.5 ppm) and showed excellent selectivity toward ethylene over a wide range of sensing temperatures (350–450 °C). The bilayer sensor used in the present study consists of a catalytic overlayer and a sensing film, and two electrodes are located beneath the sensing film. In this configuration, the responses to the analyte gases are determined by the following: gas oxidation and/or reforming in the upper catalytic layer, gas transport to the lower sensing region, and the sensing reaction between the gas and negatively charged oxygen ions on the oxide semiconductor surfaces. Limiting the diffusion of gas by coating the Cr_2_O_3_ catalytic overlayer could be excluded as a possible reason for the decrease in responses to most gases (with the exception of ethylene); this is because the Cr_2_O_3_ overlayer (thickness: ≈0.3 µm) is significantly thinner than the SnO_2_ sensing film (thickness: ≈21 µm). Therefore, it was suggested that the selective catalytic filtering of gases was the probable reason for the high ethylene selectivity; this was because the catalytic Cr_2_O_3_ overlayer barely oxidized the relatively stable ethylene, but facilitated the oxidation of the more reactive interfering gases. However, it is worth noting that the response to ethylene at 350 °C was observed to slightly increase from 12.2 to 16.8 due to the coating of the Cr_2_O_3_ overlayer (Figure 4e,f). This can be attributed to the reforming of less reactive ethylene into more reactive species by catalytic Cr_2_O_3_; this explanation is in line with the results of our previous reports, wherein the responses to less reactive aromatic gases were enhanced after the coating of oxide‐based gas sensing films with nanoscale catalytic oxide overlayers.^[^ 41, 42 ^]^ These above results clearly indicate that employing a Cr_2_O_3_ catalytic overlayer coating is a promising strategy for facilitating the highly selective and sensitive detection of ethylene.

The selectivity toward ethylene over the key interfering gases (TMA, ethanol, and HCHO) (S_Ethylene_/S_TMA_, S_Ethylene_/S_Ethanol_, and S_Ethylene_/S_HCHO_) was calculated, and results were plotted as a function of temperature (Figure S6, Supporting Information). The S_Ethylene_/S_TMA_, S_Ethylene_/S_Ethanol_, and S_Ethylene_/S_HCHO_ values increased with an increase in the thickness of the SnO_2_ sensing film (Figure S6a,b, Supporting Information); these values increased further after coating with the Cr_2_O_3_ catalytic overlayer. For the 0.3Cr_2_O_3_‐SnO_2_ sensor, all three selectivity values were maximized at 375 °C (Figure S6c, Supporting Information). Accordingly, considering both the gas selectivity and gas response, the optimal sensor operation temperature for ethylene detection was identified as 375 °C, although the higher gas response could be obtained at a lower sensing temperature (<375 °C).

**Figure 5:**
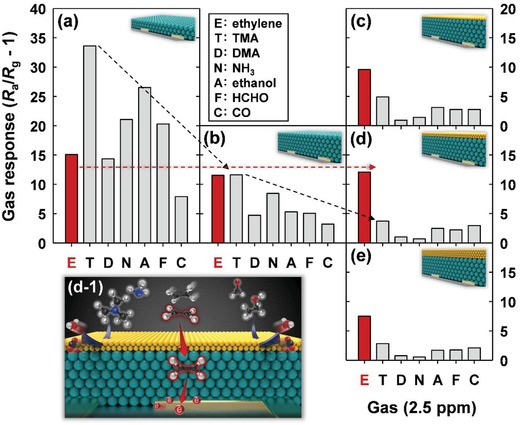
Gas‐sensing behaviors and sensing mechanism at 375 °C: a) thin SnO_2_ sensor, b) thick SnO_2_ sensor, c) 0.05Cr_2_O_3_‐SnO_2_ sensor, d) 0.3Cr_2_O_3_‐SnO_2_ sensor, and e) 0.6Cr_2_O_3_‐SnO_2_ sensor (E: ethylene; T: TMA; D: DMA; N: NH_3_; A: ethanol; F: HCHO; C: CO). The analyte gas concentration was 2.5 ppm.

The effects of the thicknesses of the SnO_2_ sensing film and Cr_2_O_3_ overlayer on the gas selectivity at 375 °C were then investigated (**Figure** **5**). Although the thickening of the SnO_2_ sensing film decreased the overall gas responses (except for ethylene), the oxidative filtering of the interfering gases due to the film thickening was insufficient for achieving ethylene selectivity (Figure 5a,b). In contrast, all the three sensors coated with 0.05‐, 0.3‐, and 0.6‐µm‐thick Cr_2_O_3_ overlayers (Figure 5c–e) exhibited excellent selectivity to ethylene, which confirmed the effectiveness of the Cr_2_O_3_ overlayers for use in the selective filtering of interfering gases. The thin (0.05 µm) Cr_2_O_3_ overlayer led to relatively high cross‐responses to interfering gases, probably because oxidation of interfering gases was less effective (Figure 5c). However, excessive thickening (0.6 µm) of the Cr_2_O_3_ overlayer reduced the responses to both ethylene and interfering gases (Figure 5e), and this could be related to the catalytic oxidation of all analyte gases, including some of the ethylene, before these were transported to the sensing film. Accordingly, the optimal thickness for the Cr_2_O_3_ overlayer was determined as 0.3 µm (Figure 5d).

To examine the effect of the Cr_2_O_3_ overlayer configuration, Cr_2_O_3_ microspheres prepared via ultrasonic spray pyrolysis were also coated on the SnO_2_ thick film (thickness: ≈19 µm), and the gas‐sensing characteristics were measured (Figure S7, Supporting Information). The Cr_2_O_3_ overlayer was ≈1 µm thick. Interestingly, the response to ethylene (*S* = 7.3) was significantly higher than that of the interfering gases (*S* = 1.1–4.3). This result again confirms that the Cr_2_O_3_ coating, regardless of the overlayer configuration, is promising for utilization in the highly selective detection of ethylene.

To elucidate the role of the bilayer sensor design used in the present study, a sensing film (thickness: ≈20 µm) comprising SnO_2_ spheres uniformly loaded with Cr_2_O_3_ was also prepared; this was achieved via loading of Cr_2_O_3_ nanoparticles on the entire surface of the SnO_2_ hollow spheres (1.0 at% Cr_2_O_3_), screen printing a slurry, and a heat treatment at 600 °C. The resulting sensor was referred to as the “Cr_2_O_3_‐loaded SnO_2_ sensor,” and its gas‐sensing characteristics were compared with those of the thick SnO_2_ and 0.3Cr_2_O_3_‐SnO_2_ sensors (Figures S8 and S9, Supporting Information). The results showed that the response of Cr_2_O_3_‐loaded SnO_2_ sensor to 2.5 ppm ethylene (*S* = 1.1) at 375 °C was significantly lower than that of the 0.3Cr_2_O_3_‐SnO_2_ sensor (*S* = 12.1). This negligibly low gas response of the Cr_2_O_3_‐loaded SnO_2_ sensor can be explained by the excessive oxidation of ethylene during its transport through the thick and highly catalytic Cr_2_O_3_‐loaded SnO_2_ film. To confirm the effect of Cr_2_O_3_ on the gas‐sensing characteristics, a Cr_2_O_3_ thin film (0.3 µm thick) was prepared via e‐beam evaporation, and its gas‐sensing characteristics were measured at 375 °C (Figure S10, Supporting Information). The pure Cr_2_O_3_ sensing film showed negligibly low gas responses to all the analyte gases, including ethylene, indicating that Cr_2_O_3_ alone cannot be used for ethylene detection. Thus, separating the catalytic and sensing reactions by using the bilayer design can provide superior controllability of the gas‐sensing characteristics.

It should be noted that the values of the sensor resistance in air (*R*_a_) for the thick SnO_2_ and 0.3Cr_2_O_3_‐SnO_2_ sensors were similar (200–300 kΩ), whereas the *R*_a_ value for the Cr_2_O_3_‐loaded SnO_2_ sensor was significantly higher (2.3 MΩ) (Figure S9, Supporting Information). This significant increase in sensor resistance for the Cr_2_O_3_‐loaded SnO_2_ sensor could be associated with aliovalent doping or the charge transfer from SnO_2_ to Cr_2_O_3_.^[^ 38 ^]^ It is also worth noting that if the sensor resistance becomes excessively high by loading the catalysts on the entire sensing materials, performing resistance measurements by using a conventional electric circuit can become difficult; this can hamper the ability to provide cost‐effective resistance measurements for actual applications. In contrast, for the present bilayer sensor design, the conduction along the lower part of the SnO_2_ sensing film near the electrodes is barely affected by the outlying Cr_2_O_3_ catalytic overlayer; this enables the control of gas‐sensing characteristics without affecting the sensor resistance. Therefore, the bilayer sensor design with the nanoscale catalytic overlayer offers various distinctive advantages, namely: an excellent gas selectivity that arises due to the oxidative filtering of interfering gases, a high gas response that arises due to the negligible limitation of gas transport, an effective separation of sensing and catalytic reactions, and the facile control of gas‐sensing characteristics without altering the sensor resistance.

The effects of the catalytic overlayer on the response and recovery kinetics of the sensor were investigated. For this, the 90% response and recovery times (τ_res_ and τ_recov_) of the sensors (the times taken to reach 90% resistance variation upon exposure to 2.5 ppm analyte gas and air) were calculated; these results are shown in Figure S11, Supporting Information. Note that the τ_res_ and τ_recov_ values for ethylene sensing were similar; this was true even after coating with the Cr_2_O_3_ catalytic overlayer; however, those values for the interfering gases were observed to decrease significantly with the Cr_2_O_3_ coating. This again indicates that the majority of the interfering gases were oxidized in the Cr_2_O_3_ catalytic overlayer and that employing the bilayer sensor design caused no deterioration in the response and recovery kinetics for ethylene sensing.

The results in Figures 4 and 5 clearly indicate that the Cr_2_O_3_ catalytic overlayer is key to enabling the selectively detecting ethylene. The nanoscale Cr_2_O_3_ overlayer coating on the thick SnO_2_ sensor caused a substantial decrease in the responses to ethanol, HCHO, and CO (Figure 5b,d). Cr_2_O_3_ exhibits multivalency, abundant oxygen adsorption, and a facile redox reaction,^[^ 43 ^]^ and is known to be an excellent catalyst for gas oxidation. For instance, Cr_2_O_3_‐loaded ZrO_2_ and Al_2_O_3_ have been used to oxidize ethanol, and mesoporous Cr_2_O_3_ is a good catalyst for oxidizing HCHO.^[^ 44 ^]^ This strongly suggests that the catalytic oxidation of ethanol, HCHO, and CO to non‐ or less‐reactive species is responsible for the low responses of 0.3Cr_2_O_3_‐SnO_2_ sensor to these three gases. It should be noted that the decrements in gas responses to amine‐containing gases (such as TMA, DMA, and NH_3_) due to the Cr_2_O_3_ overlayer coating were significantly larger than those of the responses to ethanol and HCHO (Figure 5b,d), and this could be attributed to the high chemical affinity of Cr_2_O_3_ to amine‐containing gases. For example, the basic NH_3_ molecule is known to be adsorbed easily on the Lewis acidic sites of Cr_2_O_3_ formed on surface cations (Cr^3+^).^[^ 45 ^]^ Borck et al.^[^ 46 ^]^ reported that methylamine with lone‐pair electrons on the nitrogen atom can be adsorbed favorably on the Cr_2_O_3_ surface by donating an electron to the partially empty cation orbital. Moreover, it has been reported that Cr_2_O_3_ promotes the decomposition of NH_3_^[^ 47 ^]^ and the oxidation of methyl groups in TMA and DMA.^[^ 48 ^]^ Therefore, the Cr_2_O_3_ overlayer promotes both the adsorption and oxidation of amine‐containing gases and accelerates the oxidative filtering of TMA, DMA, and NH_3_, and the substantial reductions in the responses to amine‐related gases when applying the 0.3‐µm‐thick Cr_2_O_3_ overlayer coating can be attributed to the facile adsorption of amine compounds as well as effective gas oxidation. All these findings support the fact that in the present study, the interfering gases were successfully filtered via catalytic oxidation in the nanoscale Cr_2_O_3_ overlayer, and this enabled the high selectivity to ethylene.

Ethylene is a key plant hormone, and its detection is used to determine the development and growth of climacteric fruits.^[^ 1, 2, 3, 4 ^]^ For example, it is known that a very low concentration of ethylene (0.1–1 ppm) is produced during the ripening of climacteric fruits, and that emitted ethylene can further accelerate ripening.^[^ 2, 4, 5, 6 ^]^ Accordingly, the amount of ethylene emitted from fruit can not only be employed as a useful measure to monitor fruit ripening, but it can also provide valuable information for managing fruit storage. Moreover, in “smart farming,” the ethylene concentration provides key information that is used to control the development and growth processes of plants, such as seed germination, flowering, leaf abscission, fruit formation, and senescence. In such an industry, it is essential to precisely detect and manage low concentrations of ethylene. However, the responses of metal oxide semiconductor gas sensors to ethylene are generally significantly lower than the responses to more reactive interfering gases, including food‐related gases (TMA, DMA, and NH_3_), ubiquitous ethanol, and indoor air pollutants (HCHO and CO).^[^ 29, 30, 31, 32, 49, 50 ^]^ Accordingly, it is very challenging to detect ethylene in a highly selective and sensitive manner in the presence of the aforementioned interfering gases.

**Figure 6:**
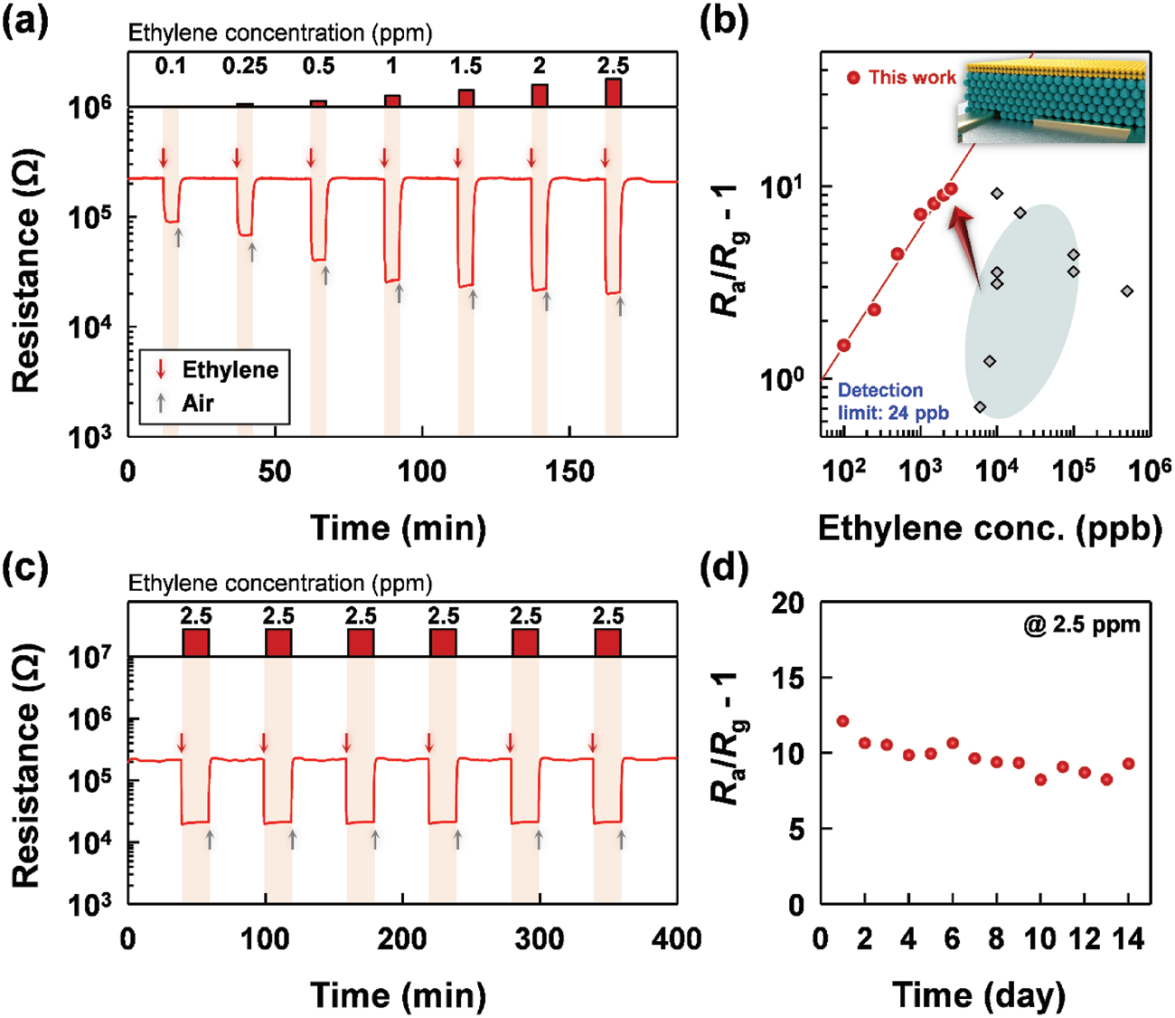
a) Dynamic gas‐sensing transients of the 0.3Cr_2_O_3_‐SnO_2_ sensor exposed to 0.1–2.5 ppm ethylene at 375 °C; b) gas response as a function of ethylene concentration and ethylene response reported in literature;^[^ 25, 27, 28, 29, 30, 47, 48, 49 ^]^ c) six repeated measurements of the sensing properties of the sensor to 2.5 ppm ethylene at 375 °C; d) long‐term stability of the sensor over 15 days.

As previously mentioned, many attempts have been made to enhance ethylene sensing properties, for example, using WO_3_‐loaded SnO_2_ nanoparticles,^[^ 29 ^]^ Pt‐doped SnO_2_ nanoparticles,^[^ 30 ^]^ Pd/rGO‐loaded Fe_2_O_3_ hierarchical nanostructures,^[^ 31 ^]^ and Pd‐doped SnO_2_ nanoparticles.^[^ 32 ^]^ However, most of these sensors exhibited relatively low gas responses (*S* < 9) to high concentrations of ethylene (ranging from 10–100 ppm). The sensing transients of the 0.3Cr_2_O_3_‐SnO_2_ sensor to 0.1–2.5 ppm ethylene at 375 °C were measured (**Figure** **6**a). The detection limit of ethylene was estimated to be as low as 24 ppb when *R*_a_/*R*_g_ − 1 > 1.2 was used as the criterion for sensing (Figure 6b). The ethylene response of the 0.3Cr_2_O_3_‐SnO_2_ sensor is among the highest ever reported in literature (**Table** **1**).^[^ 27, 29, 30, 31, 32, 51, 52, 53 ^]^ The relatively low ethylene responses of CNT‐based sensors can be explained by the insufficient thermal activation of gas sensing reaction due to the low operation temperature (25–150 °C).^[^ 27, 53 ^]^ The ethylene response in the present sensor is also superior to those of other sensors using catalyst‐loaded SnO_2_ nanoparticles with similar or higher operational temperature (≥ 350 °C) in the literature.^[^ 30, 32, 52 ^]^ This can be attributed to the effective separation and tailoring of the catalytic and sensing reaction using bilayer design as well as highly gas accessible sensing materials with hollow morphology. The sensor exhibited highly reproducible sensing and recovery upon repeated exposure to 2.5 ppm ethylene and showed good long‐term stability over 15 days (Figure 6c,d). The ethylene selectivity of the 0.3Cr_2_O_3_‐SnO_2_ sensor in the presence of different concentrations of interfering gases (TMA and ethanol) was also examined. The response to 2.5 ppm ethylene was barely affected by the coexistence of 0.01–2.5 ppm TMA or ethanol (Figure S12, Supporting Information), which indicates that this sensor can be used in real applications in mixed gas environments.

**Table 1:** Gas responses (*R*_a_ *R*_g_^−1^ − 1, *R*_g_ *R*_a_^−1^ − 1, *I*_g_ *I*_a_^−1^ − 1) of various materials to ethylene, as reported in literature and obtained in the present study^[^ 27, 29, 30, 31, 32, 51, 52, 53 ^]^

Materials	Conc. [ppm]	Response (*R*_a_ *R*_g_^−1^ − 1, *R*_g_ *R*_a_^−1^ − 1, *I*_g_ *I*_a_^−1^ − 1)	Sensor temp. [°C]	τ_res_ */τ*_recov_ [s]	Ref.
Pd‐doped SnO_2_ nanoparticles	100	3.53	450	—	^[^ 30 ^]^
Au‐doped SnO_2_ nanoparticles	100	4.33	450	—	^[^ 30 ^]^
Pt‐doped SnO_2_ nanoparticles	20	7.13	350	—	^[^ 32 ^]^
Pd/rGO‐loaded Fe_2_O_3_ nanostructures	10	9	250	18/50	^[^ 31 ^]^
WO_3_ platelets	10	3.5	500	—	^[^ 51 ^]^
Silicalite‐coated SnO_2_ thin films	10	1.21	350	14/144	^[^ 52 ^]^
WO_3_‐loaded SnO_2_ nanoparticles	6	0.7	300	—	^[^ 29 ^]^
Cu‐added CNTs	50	0.018	RT	—	^[^ 27 ^]^
B‐doped CNTs	30	0.0011	150	—	^[^ 53 ^]^
0.3Cr_2_O_3_‐SnO_2_ hollow spheres	2.5	16.8	350	6/69	This work
0.3Cr_2_O_3_‐SnO_2_ hollow spheres	2.5	12.1	375	9/69	This work

**Figure 7:**
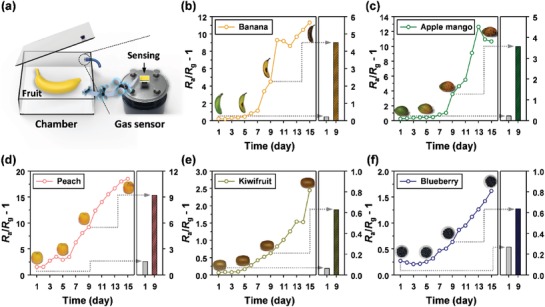
a) Schematic of fruit measurement system. Gas‐sensing characteristics of the 0.3Cr_2_O_3_‐SnO_2_ sensor for five different fruit types: b) banana, c) apple mango, d) peach, e) kiwifruit, and f) blueberry.

To evaluate the potential of the 0.3Cr_2_O_3_‐SnO_2_ sensor for assessing fruit freshness, we investigated time‐dependent changes in the ethylene concentration emitted from various fruit types (banana, apple mango, peach, kiwifruit, and blueberry) with similar weights (≈100 g) (**Figure** **7**). For this, the five different fruit types were stored in open air (relative humidity of 35–55%) at room temperature (20–25 °C) without any treatment, and the changes in the peel color and the concentration of emitted ethylene were monitored over 15 days (Figure 7; Figure S13, Supporting Information). Measurements were conducted using the following procedures: when sensor resistance became constant in the air atmosphere, the fruit was enclosed in an acrylic chamber with a fixed volume (inner volume: 10 cm × 10 cm × 5 cm) for 10 min (Figure 7a), and sensor resistance was then measured in the presence of the fruit. Ethylene responses of the sensor to the banana, apple mango, peach, kiwifruit, and blueberry on the first day were 0.22, 0.22, 1.54, 0.06, and 0.27, respectively, and these different ethylene responses suggest that the sensor can be used to discriminate between fruits. The ethylene responses for all the climacteric fruit increased gradually during fruit ageing in an ambient atmosphere, which indicated an increase in the ethylene concentration.^[^ 54, 55 ^]^ This clearly demonstrates that the ethylene sensor can be used to assess the freshness and ripening of fruits. It is of note that banana, apple, and mango changed color with ripening, whereas the peach, kiwifruit, and blueberry showed no notable color variations. In this respect, it is important to know the ethylene concentration to assess the freshness of fruit that does not undergo a color change. Moreover, people select their fruit in accordance with certain and complex criteria, such as color, gloss, hardness, and scent, and these criteria can be closely correlated with ethylene concentrations. Accordingly, quantitative measurements of ethylene concentrations can facilitate the selection of high‐quality fruit.

In actual applications, fruit is often stored with meat or fish, which emit biogenic amines such as TMA, DMA, and NH_3_. To examine the cross‐responses of the Cr_2_O_3_‐SnO_2_ bilayer sensor to biogenic amines, we conducted an ethylene recognition test for a banana in the absence and presence of seafood and/or meat, and the results are shown in Figure S14 and Video S1, Supporting Information. The response of the 0.3Cr_2_O_3_‐SnO_2_ sensor to the banana was barely affected by the introduction of seafood and/or meat. These results clearly demonstrate that the proposed sensor can be used to monitor the freshness of fruit in the presence of other foods (e.g., in a refrigerator).

The effect of ambient humidity on the gas sensing characteristics, which is also important for practical applications, was investigated by measuring the sensing transients upon exposure to the same banana under different humidity conditions in an acrylic chamber (inner volume: 40 cm × 30 cm × 12 cm) containing a miniaturized humidifier (Figure S15, Supporting Information). The decrease of sensor resistance and gas response was observed with an increase in humidity from r.h. 21% to r.h. 37%. The former is associated with the generation of electrons due to the reaction between ionized oxygen on the surface and water vapor, and the latter is attributed to the decrease of ionized oxygen available for gas sensing due to formation of surface hydroxyl groups.^[^ 56, 57 ^]^ The humidity‐dependent changes in sensor resistance and gas response in this study are consistent with those present in literature.^[^ 16 ^]^ Note that the sensor showed the similar and high responses to a banana under different humidity conditions, indicating that the present sensor is suitable for use in humid conditions or conditions in which humidity changes.

**Figure 8:**
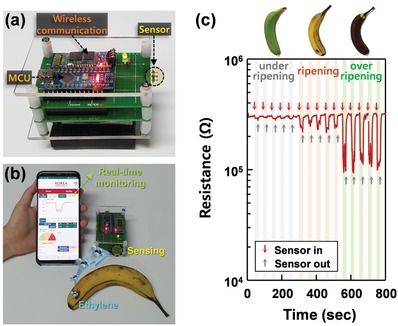
a) Wireless sensor module used to assess and monitor fruit freshness. b) Photograph of gas‐sensing test. c) Real‐time fruit freshness monitoring using sensing module wirelessly connected to a smartphone.

To demonstrate the potential of the Cr_2_O_3_‐SnO_2_ bilayer sensor for use in real‐time and onsite fruit freshness monitoring, a portable sensing module wirelessly connected to a mobile phone was developed (**Figure 8**a,b). When conducting measurements, the distance between the sensing part (dotted circle in Figure 8a) and a banana was varied in the range of ≈0.5 to ≈15 cm, and the analog sensing signals were processed by the analog‐to‐digital converter of a micro‐controller unit (MCU, ATmega328, Microchip Technology Inc., USA) and then transmitted to a smartphone via Bluetooth communication. The sensor successfully discriminated three bananas with different levels of ripening (Figure 8c; Video S2, Supporting Information) and showed highly reproducible and reversible sensing characteristics, which thus confirms its promising potential for use in assessing the freshness and ripening of fruits in real applications. In summary, the proposed Cr_2_O_3_‐SnO_2_ bilayer sensor exhibits ultrahigh selectivity, high sensitivity, rapid sensing kinetics, good stability, and negligible interference from seafood/meat. The significant breakthroughs presented in this paper will open various pathways for applying this sensor in a wide range of agricultural applications that benefit from plant‐hormone control.

## 3 Conclusion

In this study, a novel bilayer sensor with a SnO_2_ sensing layer and a nanoscale catalytic Cr_2_O_3_ overlayer is developed, which acts as a promising solution for facilitating the exclusive detection of sub‐ppm‐levels of ethylene (the representative gas hormone of plants). The SnO_2_ sensors with the Cr_2_O_3_ overlayer exhibit ultrahigh selectivity and responses to ethylene, whereas SnO_2_ sensors without the overlayer showed similar responses to a variety of gases. The exceptional sensing properties, such as ultra‐selectivity and highly sensitive ethylene detection, are attributed to the oxidation of food‐related interfering gases (TMA, DMA, NH_3_), ubiquitous ethanol, and indoor pollutants (HCHO and CO) into less‐ or non‐ reactive species by the nanoscale catalytic Cr_2_O_3_ overlayer. In addition, the bilayer sensor design enables the sensing and catalytic reactions to be separated into independent processes, which allows gas selectivity to be precisely controlled. The outlying nano‐thin catalytic overlayer is advantageous for avoiding the limitation of gas transport to the sensing region and for preventing significant sensor resistance changes up to an unmeasurable range, which are often observed when a sensor is uniformly loaded with a catalyst. The exclusive ethylene detection capability of the unique bilayer sensor enables the monitoring of the ripening of five different climacteric fruit types in a precise and quantitative manner, and its development can be utilized in a wide range of agricultural applications that benefit from the plant‐hormone control.

## 4 Experimental Section

##### Synthesis of SnO_2_ Hollow Spheres

SnO_2_ hollow spheres were prepared via one‐pot ultrasonic spray pyrolysis (Figure S16, Supporting Information). The spray‐pyrolysis system consisted of six piezoelectric droplet generators (resonance frequency: 1.7 MHz), a tubular reactor, and a powder‐collecting chamber. Tin (II) chloride dihydrate (SnCl_2_∙2H_2_O, ≥98%, Sigma–Aldrich, USA), citric acid monohydrate (C_6_H_8_O_7_∙H_2_O, ≥99.0%, Sigma–Aldrich, USA), and a diluted hydrochloric acid solution (35.0–37.0%, HCl: distilled water = 1:99 by vol%) (4 mL) were dissolved in 200 mL of distilled water, with stirring for 1 h. The concentrations of tin chloride and citric acid were 0.1 and 0.25 m, respectively. Large quantities of droplets were generated by ultrasonic transducers and these were transferred into a high‐temperature (700 °C) tubular quartz reactor (inner diameter: 50 mm; length: 1200 mm) by air at a flow rate of 20 L·min^−1^. The Sn‐containing precursor powder was collected through a Teflon bag filter in a particle‐collection chamber and was converted into SnO_2_ hollow spheres via heat treatment at 600 °C for 2 h.

##### Preparation of SnO_2_ Sensing Film and Catalytic Cr_2_O_3_ Overlayer

The slurry for the film coating was prepared by mixing SnO_2_ hollow spheres with a terpineol‐based ink vehicle (FCM, USA) at a ratio of 1:3 (by weight). The sensing film was screen‐printed on alumina substrates (area: 1.5 mm × 1.5 mm; thickness: 0.25 mm) with two Au electrodes on the upper surface (electrode gap: 0.2 mm) and a micro‐heater on the lower surface. The thicknesses of the SnO_2_ films were controlled by changing the emulsion thickness of the screen mask. After screen printing, the sensing film was heat‐treated at 450 °C for 3 h to remove organic components. Subsequently, catalytic Cr_2_O_3_ overlayers were deposited on the SnO_2_ sensing film via the e‐beam evaporation of polycrystalline Cr_2_O_3_ grains (99.7%, Kojundo Chemical Laboratory, Japan) in a vacuum environment (base pressure = 2 × 10^−6^ Torr).

##### Characterization

Field‐emission SEM (SU‐70, Hitachi Co. Ltd., Japan) was conducted to examine the morphologies and microstructures of the materials and sensing films. The composition of the SnO_2_ film coated with a Cr_2_O_3_ overlayer was investigated using field‐emission EPMA (JXA‐8530F, JEOL Co. Ltd., Japan). HR‐TEM and elemental‐mapping images of the SnO_2_ and Cr_2_O_3_‐coated SnO_2_ nanospheres were obtained using JEM‐ARM200F (JEOL, Co. Ltd., Japan). For the TEM analysis, the spheres were detached from the uppermost region of the sensing film, dispersed in an ethanol solution, and subsequently deposited onto Cu grids (HC300‐Cu, Electron Microscopy Sciences, USA). The phases and crystal structures were investigated using X‐ray diffraction (D/MAX‐2500 V/PC, Rigaku, Japan) with CuKα radiation (λ = 1.5418 Å), and the surface area and pore‐size distribution of the SnO_2_ hollow spheres were analyzed using N_2_ adsorption via the BET method (TriStar 3000, Micromeritics, USA)

##### Gas‐Sensing Characteristics

The sensors were heated at 500 °C for 2 h for stabilization prior to measurement. They were placed in a quartz tube (inner volume: 1.5 cm^3^), and the gas atmosphere was controlled using an automatic four‐way valve. The flow rate of the gas was fixed at 50 cm^3^·min^−1^. The direct‐current two‐probe resistance was measured using an electrometer (6487 picoammeter/voltage source, Keithley, USA) interfaced with a computer. The analyte gas concentration was controlled by changing the mixing ratio between the analyte gas and synthetic air. The sensing transients to 2.5 ppm ethylene, TMA, DMA, NH_3_, ethanol, HCHO, and CO were measured at 350–450 °C in a dry atmosphere (Figure S17, Supporting Information).

## Conflict of Interest

The authors declare no conflict of interest.

## Supporting information

Supporting Information

Supplemental Video 1

Supplemental Video 2
