# Supplementary material for: A New Strategy for Detecting Plant Hormone Ethylene Using Oxide Semiconductor Chemiresistors: Exceptional Gas Selectivity and Response Tailored by Nanoscale Cr2O3 Catalytic Overlayer
Source: Adv Sci (Weinh). 2020 Feb 24;7(7):1903093. doi: 10.1002/advs.201903093 (PMC7141008; doi:10.1002/advs.201903093)
Supplement: Supplementary file 1 — Supporting Information [file ADVS-7-1903093-s001.pdf]

Copyright WILEY-VCH Verlag GmbH & Co. KGaA, 69469 Weinheim, Germany, 2020.

ADVANCED SCIENCE Open Access

Supporting Information

for *Adv. Sci.,* DOI: 10.1002/advs.201903093

A New Strategy for Detecting Plant Hormone Ethylene Using Oxide Semiconductor Chemiresistors: Exceptional Gas Selectivity and Response Tailored by Nanoscale Cr_2O_3 Catalytic Overlayer

*Seong-Yong Jeong, Young Kook Moon, Tae-Hyung Kim, Sei-Woong Park, Ki Beom Kim, Yun Chan Kang, and Jong-Heun Lee**

Copyright WILEY-VCH Verlag GmbH & Co. KGaA, 69469 Weinheim, Germany, 2019.

Supporting Information

A new strategy for detecting plant hormone ethylene using oxide semiconductor chemiresistors: Exceptional gas selectivity and response tailored by nanoscale Cr_2O_3 catalytic overlayer

*Seong-Yong Jeong, Young Kook Moon, Tae-Hyung Kim, Sei-Woong Park, Ki Beom Kim, Yun Chan Kang, and Jong-Heun Lee**

S.-Y. Jeong, Y. K. Moon, T.-H. Kim, S.-W. Park, K. B. Kim,

Prof. Y. C. Kang, Prof. J.-H. Lee.

Department of Materials Science and Engineering, Korea University,

Seoul 02841, Republic of Korea.

E-mail: jongheun@korea.ac.kr

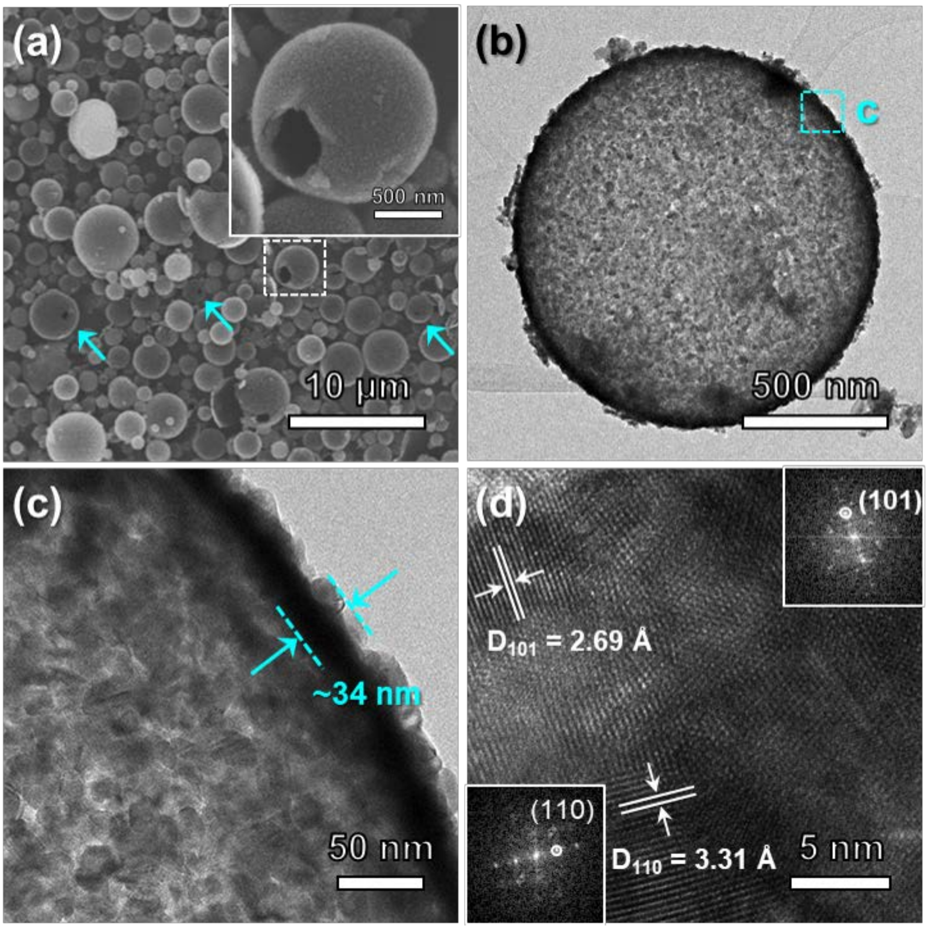

Figure S1. (a) SEM and (b-d) TEM images of SnO_2 hollow spheres.

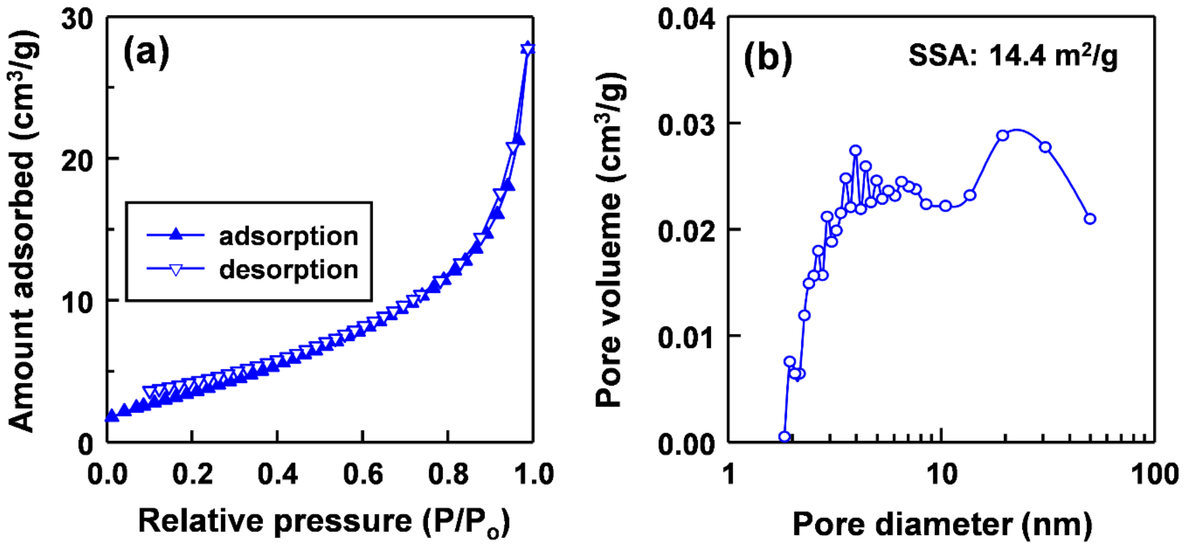

Figure S2. (a) N_2 adsorption/desorption isotherm and (b) pore-size distribution of SnO_2 hollow spheres (SSA: BET specific surface area).

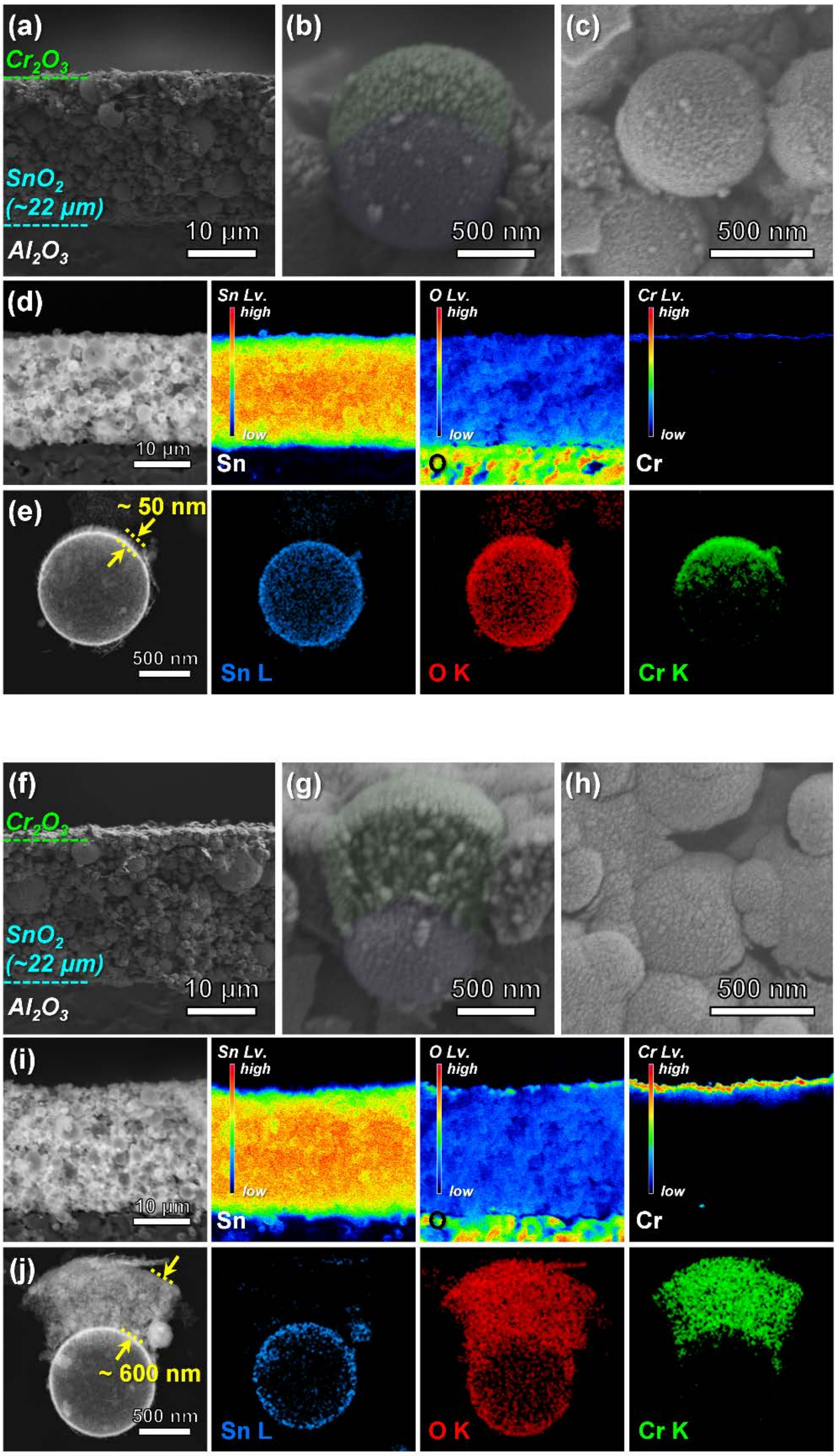

Figure S3. Microstructure and morphology characterization of the $0.05Cr_2O_3$-SnO_2 sensor and $0.6Cr_2O_3$-SnO_2 sensor: (a, f) cross-sectional SEM image of the entire sensing film, (b, g) high-magnification SEM image of the uppermost region of the sensing film, (c, h) top-view image, (d, i) backscattered image and EPMA elemental (Sn, O, and Cr) mapping and (e, j) backscattered image and TEM elemental (Sn, O, and Cr) mapping of a sphere detached from the uppermost region of the sensing film.

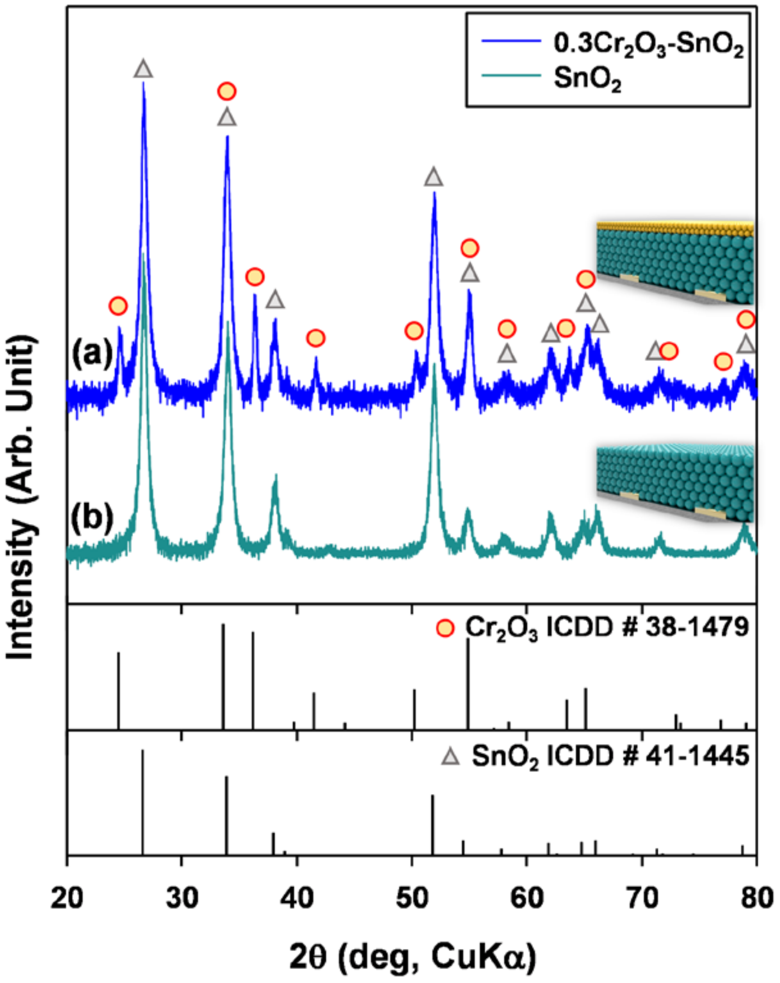

Figure S4. XRD patterns of (a) $0.3Cr_2O_3$-SnO_2 sensor, and (b) SnO_2 sensor.

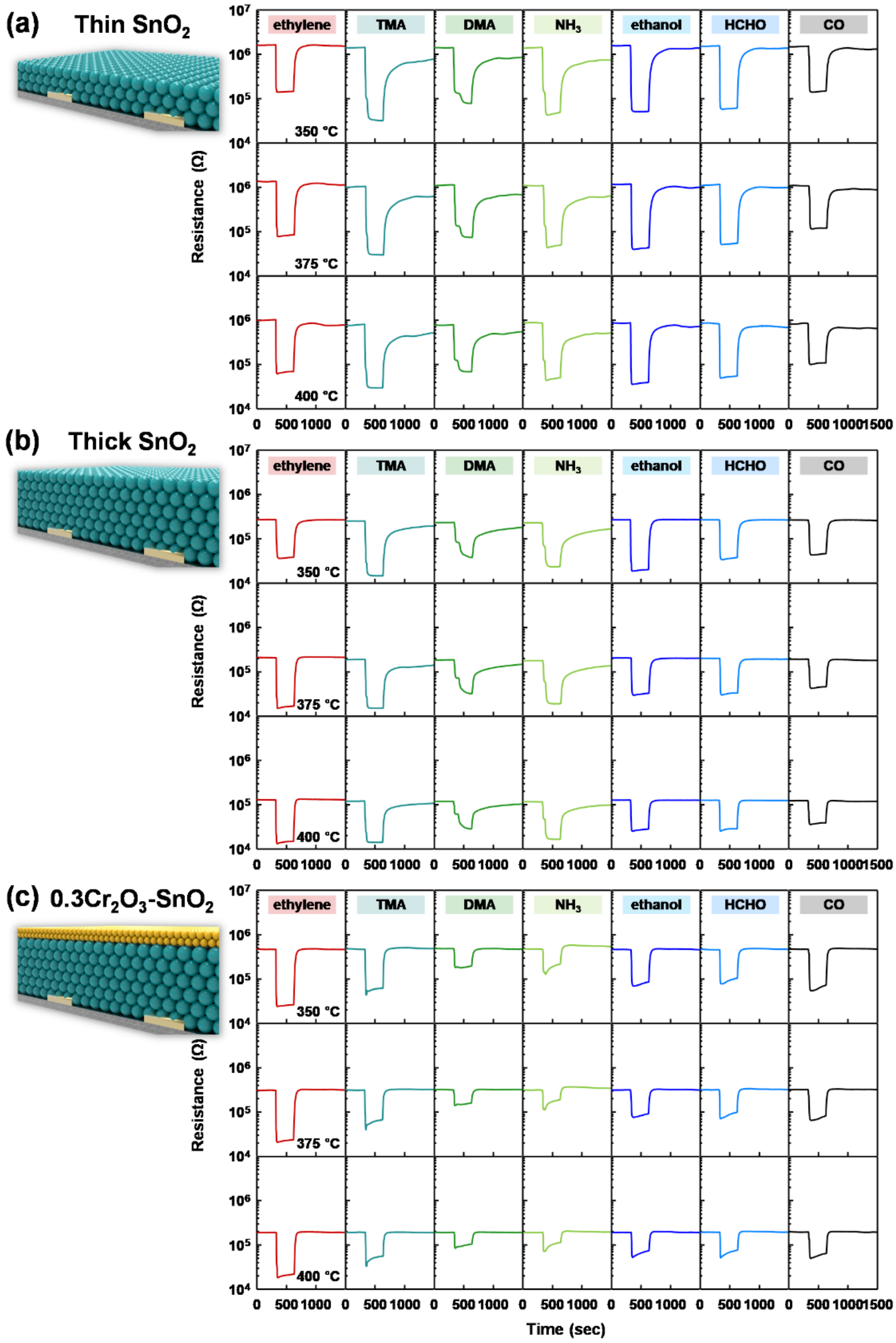

Figure S5. Dynamic sensing transients of (a) thin SnO_2 sensor (thickness: ~9 μm), (b) thick SnO_2 sensor (thickness: ~22 μm), and (c) $0.3Cr_2O_3$-SnO_2 sensor (thickness: ~21 μm) (concentration of the analyte gas: 2.5 ppm; temperature range: 350–450 °C).

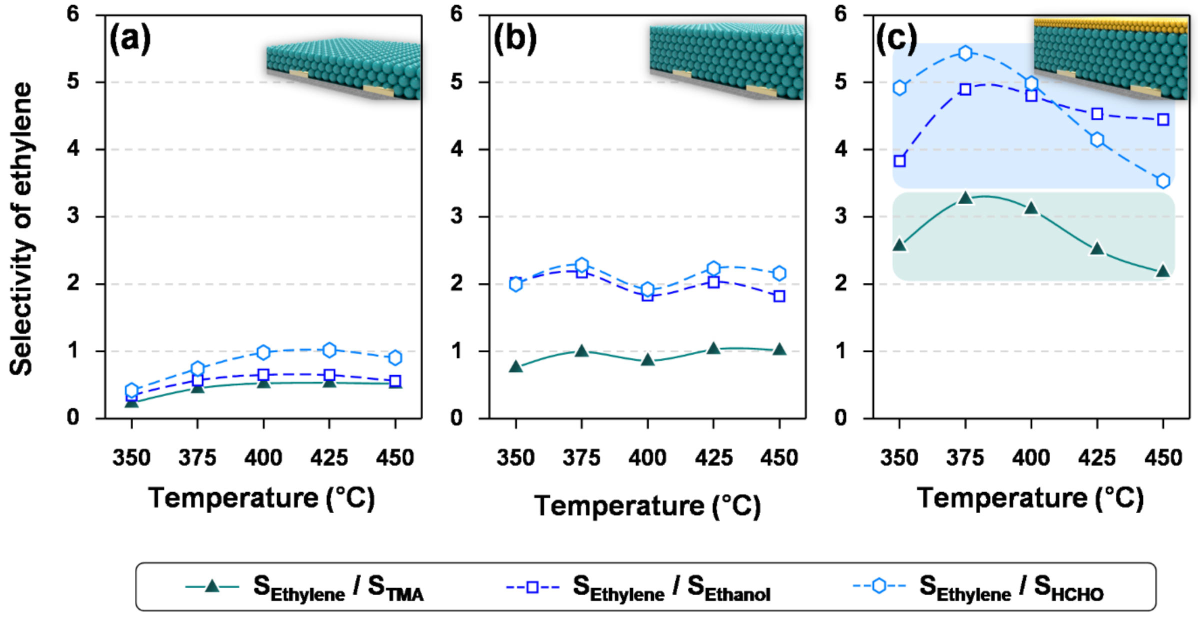

Figure S6. Ethylene selectivity ($S_{Ethylene}$ / S_{TMA}, $S_{Ethylene}$ / $S_{Ethanol}$, $S_{Ethylene}$ / S_{HCHO}) of the (a) thin SnO_2 sensor, (b) thick SnO_2 sensor, and (c) 0.3Cr_2O_3-SnO_2 sensor (concentration of the analyte gas: 2.5 ppm; temperature range: 350–450 °C).

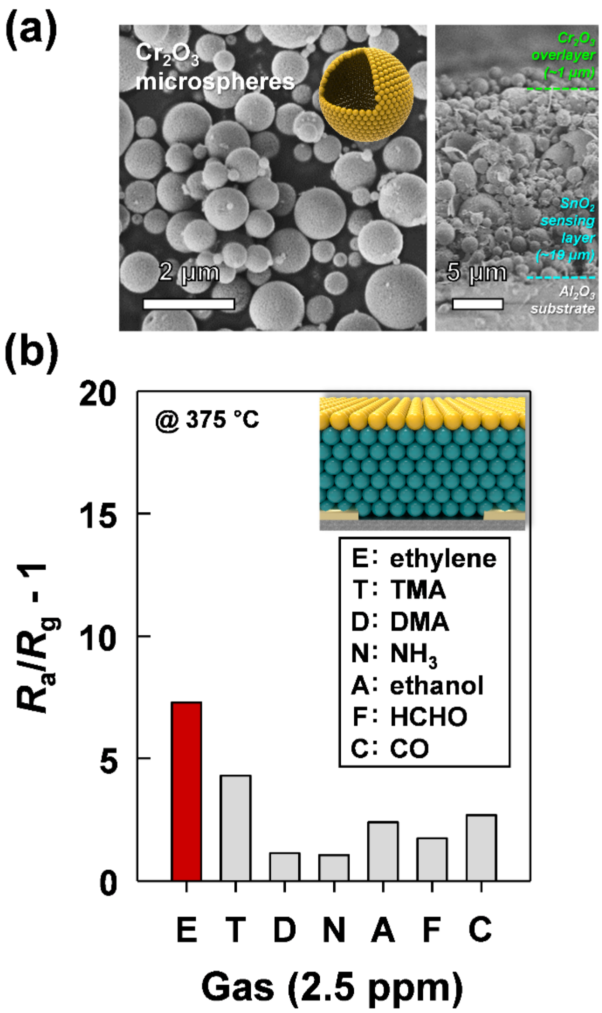

Figure S7. (a) SEM images of Cr_2O_3 microspheres, (b) cross-sectional SEM image and (b) gas-sensing properties of Cr_2O_3 microspheres-coated SnO_2 sensor at 375 °C (E: ethylene; T: TMA; D: DMA; N: NH_3; A: ethanol; F: HCHO; C: CO). The concentration of the analyte gas was 2.5 ppm.

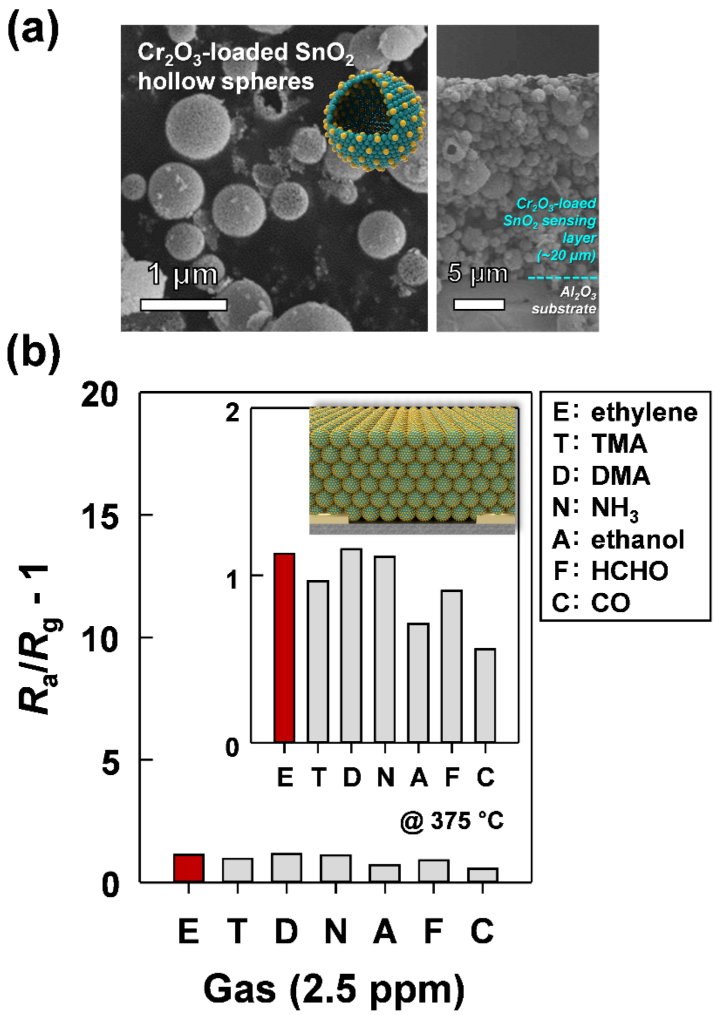

Figure S8. (a) SEM images and (b) gas-sensing properties of Cr_2O_3-loaded SnO_2 hollow spheres at 375 °C (E: ethylene; T: TMA; D: DMA; N: NH_3; A: ethanol; F: HCHO; C: CO). The concentration of the analyte gas was 2.5 ppm.

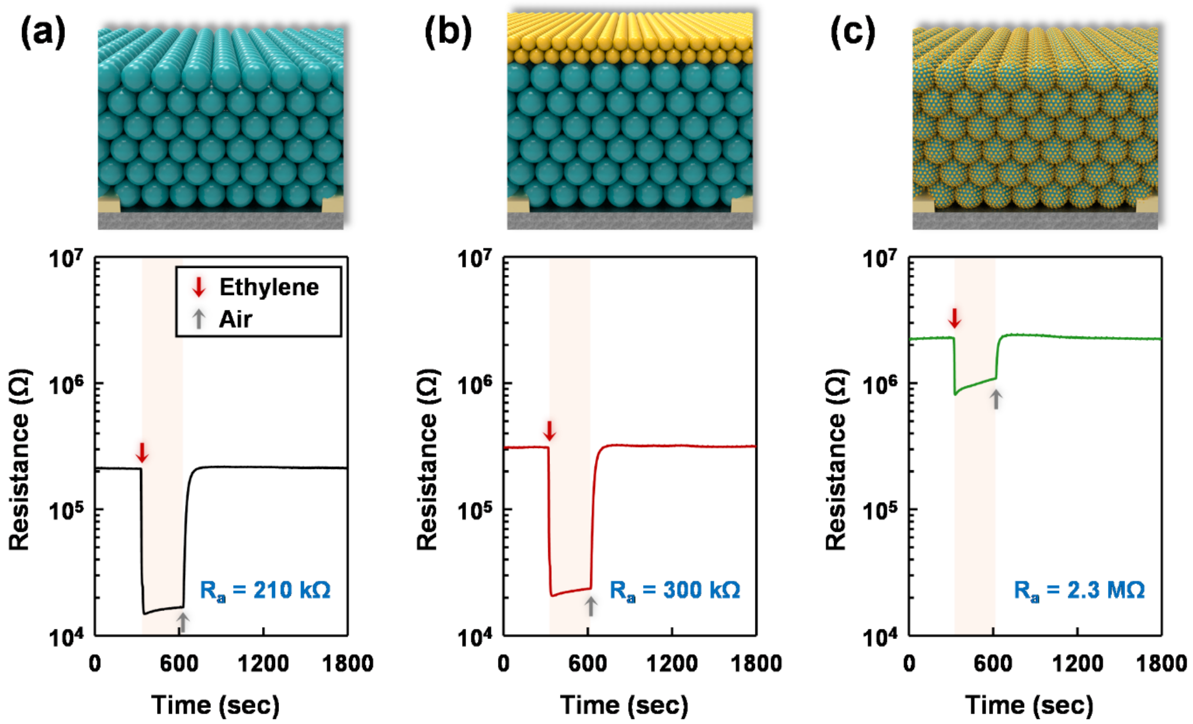

Figure S9. Schematic illustration and dynamic sensing transients of the (a) thick SnO_2 sensor, (b) 0.3Cr_2O_3-SnO_2 sensor, and (c) SnO_2 sensor uniformly loaded with 1.0 at% Cr_2O_3 (sensing temperature: 375 °C).

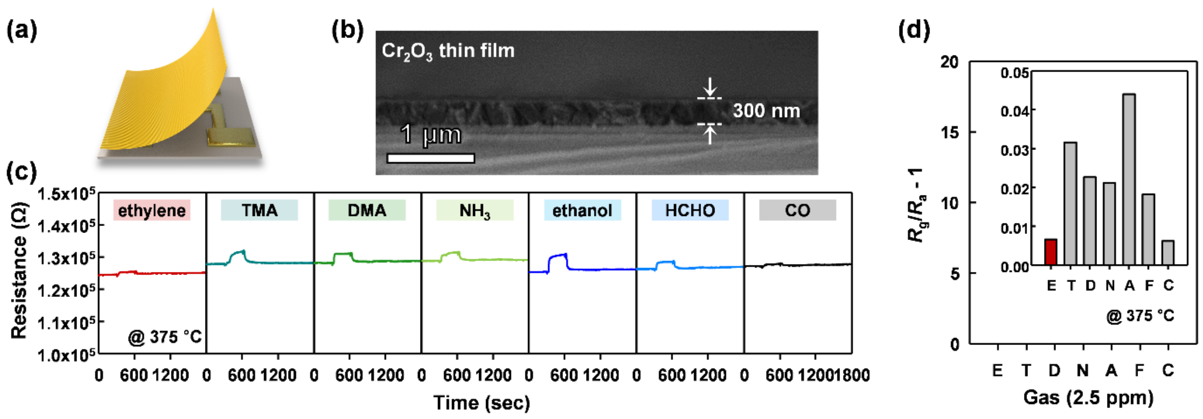

Figure S10. (a) Schematic illustration, (b) SEM image, (c) dynamic sensing transients, and (d) gas-sensing properties of the Cr_2O_3 thin film sensor at 375 °C (E: ethylene; T: TMA; D: DMA; N: NH_3; A: ethanol; F: HCHO; C: CO). The concentration of the analyte gas was 2.5 ppm.

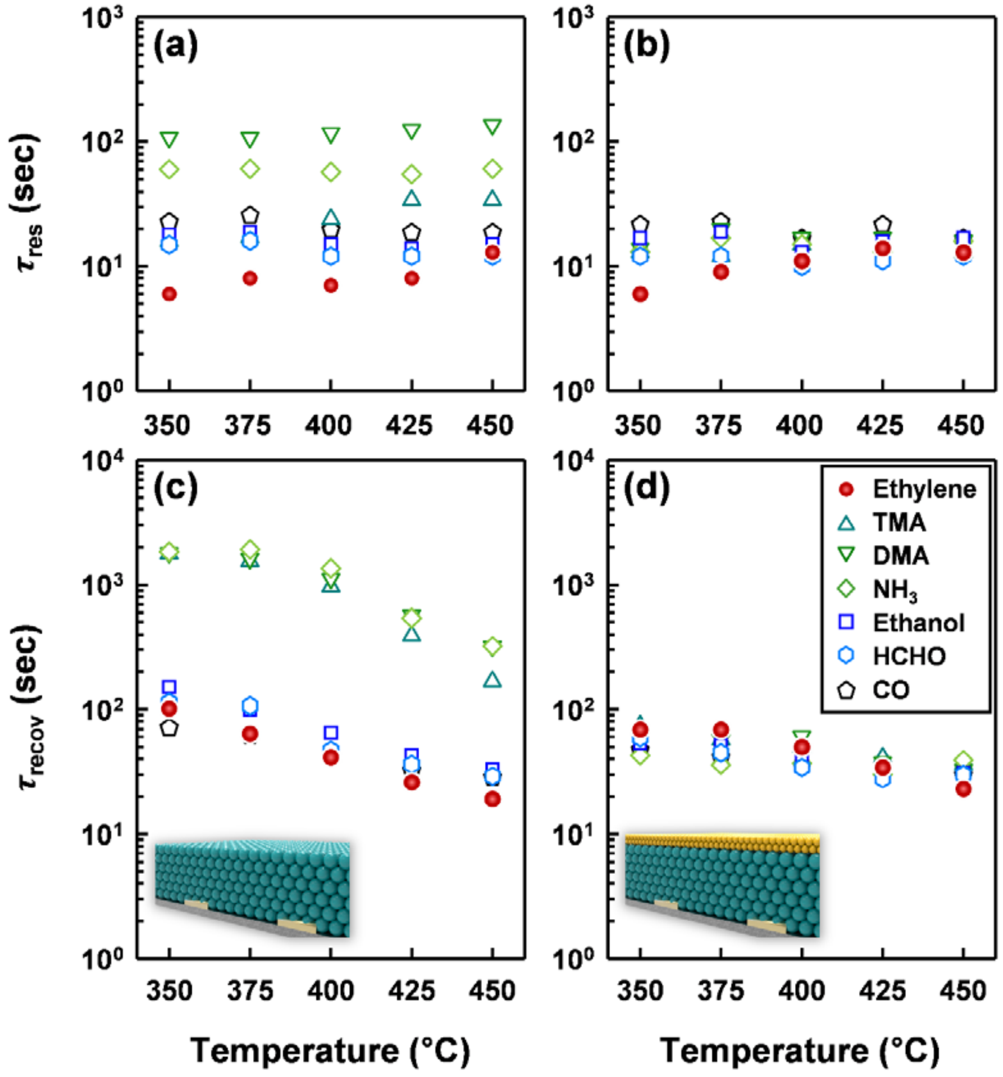

Figure S11. 90% response time (τ_{res}) and 90% recovery time (τ_{recov}) of the (a,c) thick SnO_2 sensor and (b,d) $0.3Cr_2O_3$-SnO_2 sensor at temperatures in the range of 350–450 °C.

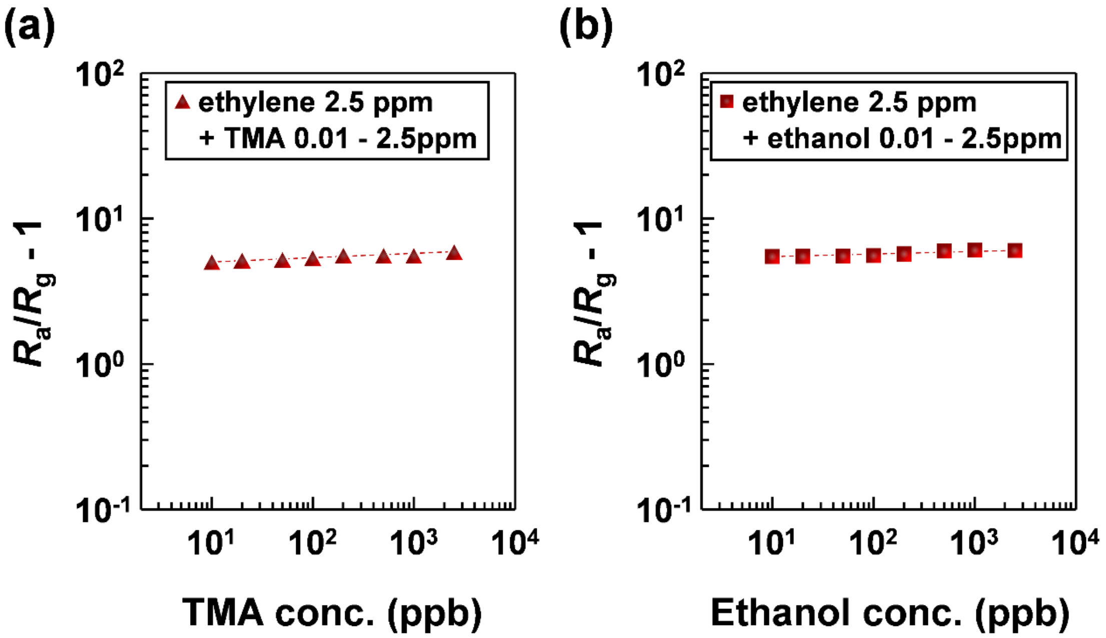

Figure S12. Gas sensing characteristics of the 0.3Cr_2O_3-SnO_2 sensor to (a) gas mixture of 2.5 ppm ethylene and 0.01–2.5 ppm TMA, (b) gas mixture of 2.5 ppm ethylene and 0.01–2.5 ppm ethanol at 375 °C.

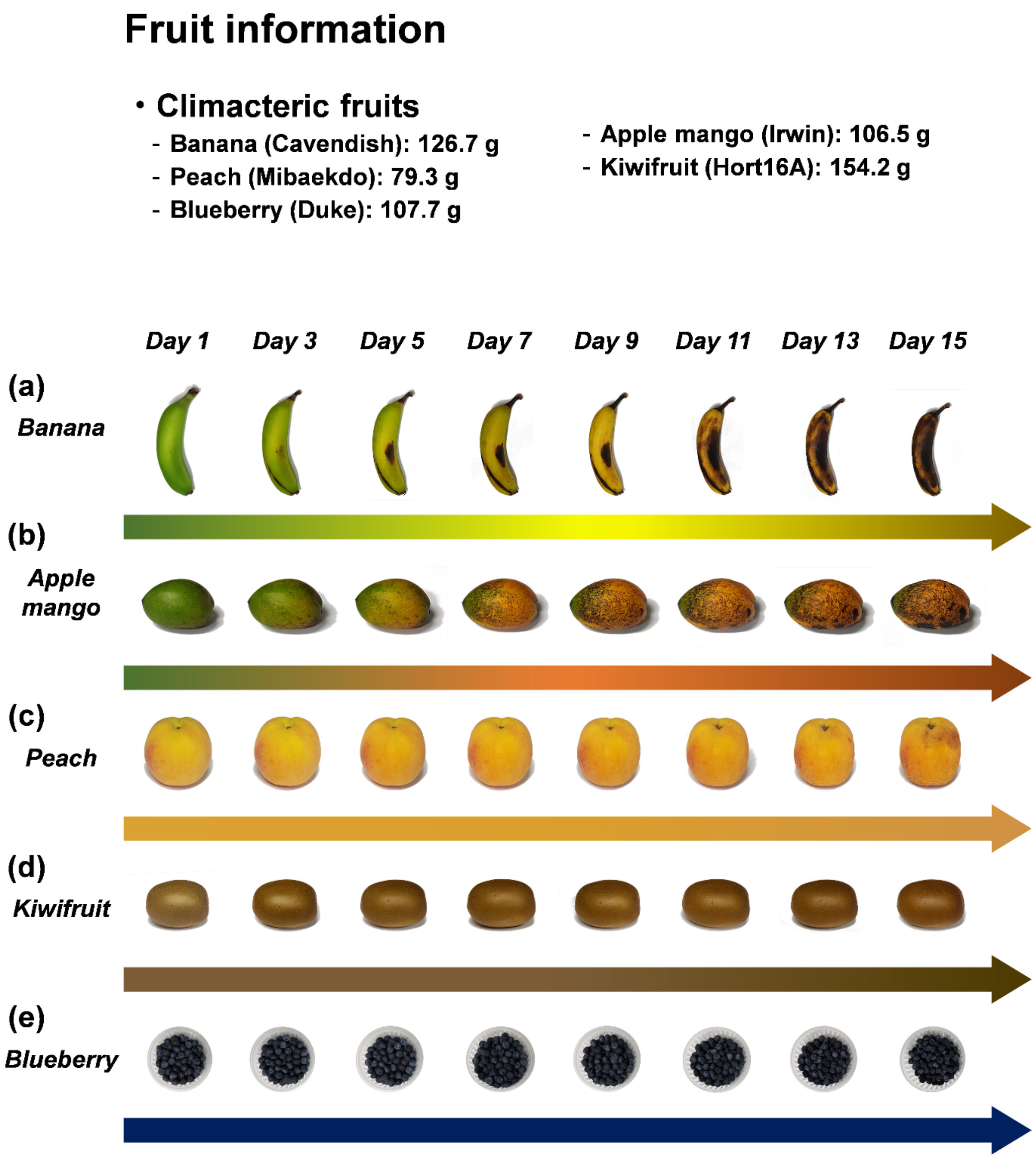

Figure S13. Information and peel color changes of five different fruits over 15 days: (a) banana, (b) apple mango, (c) peach, (d) kiwifruit, and (e) blueberry.

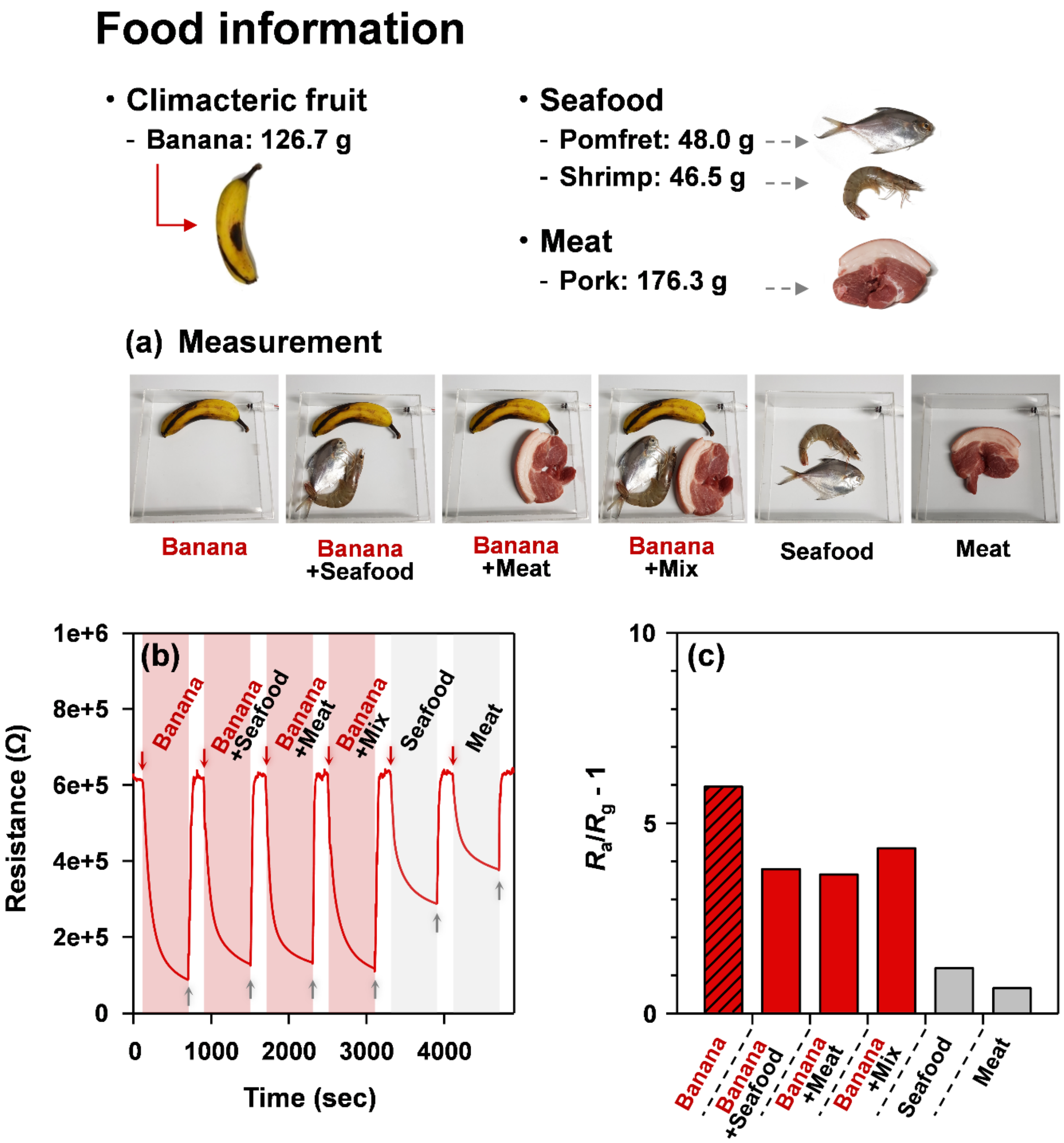

Figure S14. Information of foods and gas responses of the 0.3Cr_2O_3-SnO_2 sensor to banana mixed with other foods including seafood and/or meat. An acrylic chamber with a fixed volume (inner volume: 10 cm × 10 cm × 5 cm) was used for measuring gas sensing characteristics.

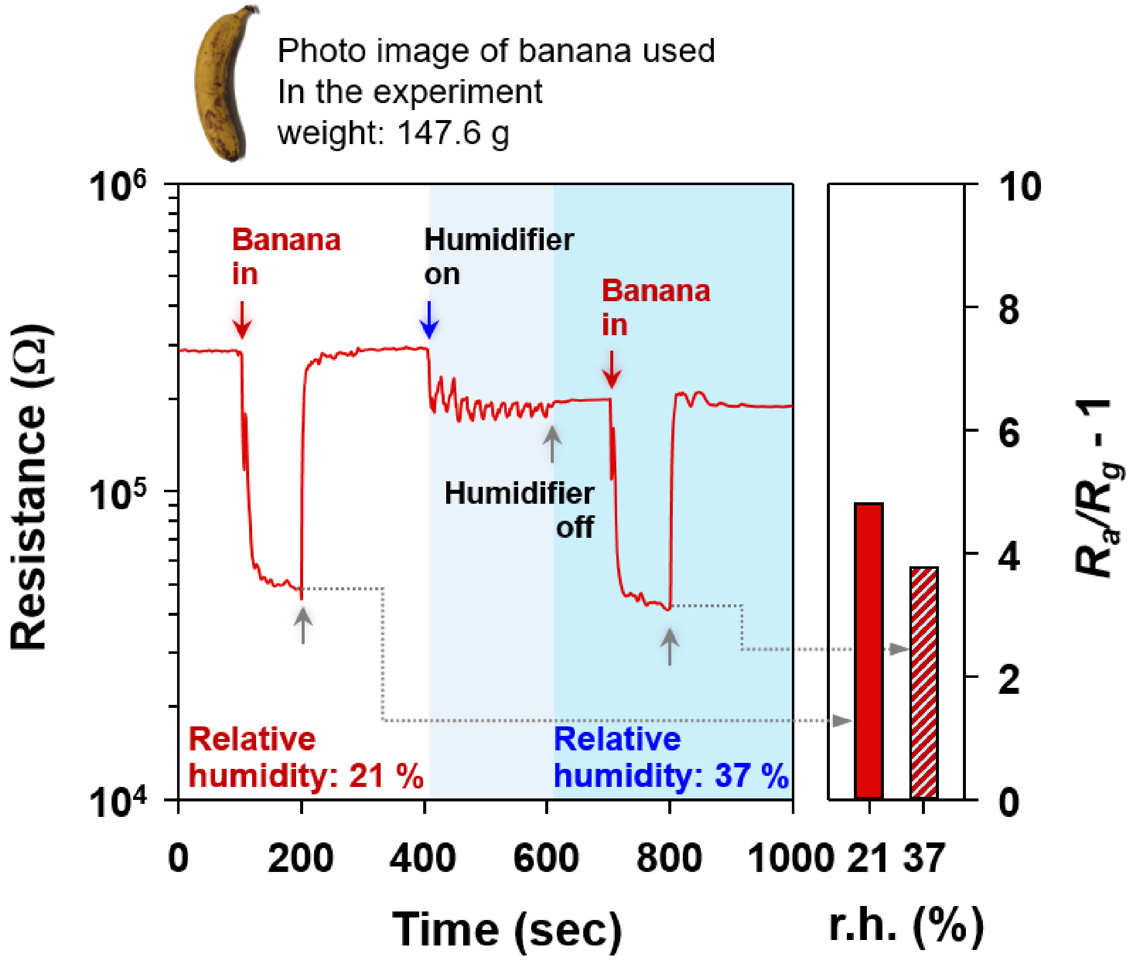

Figure S15. Gas sensing characteristics of the $0.3Cr_2O_3$-SnO_2 sensor to banana under different humidity conditions (relative humidity: 21 % and 37 %). An acrylic chamber with a fixed volume (inner volume: 40 cm x 30 cm x 12 cm) was used for measuring gas sensing characteristics. The humidity in the chamber was controlled by miniaturized humidifier. The ambient temperature was 18 °C and the sensor temperature was 375 °C.

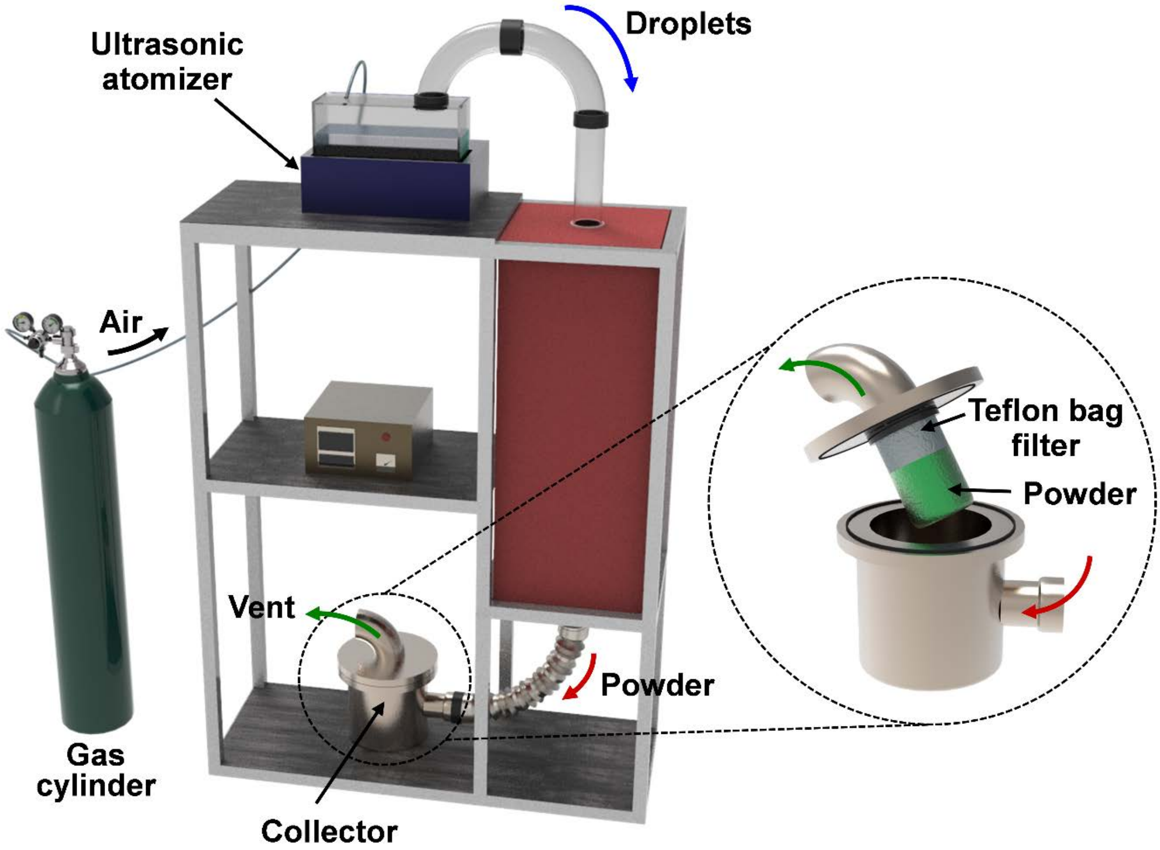

Figure S16. Schematic illustration of the ultrasonic spray pyrolysis setup.

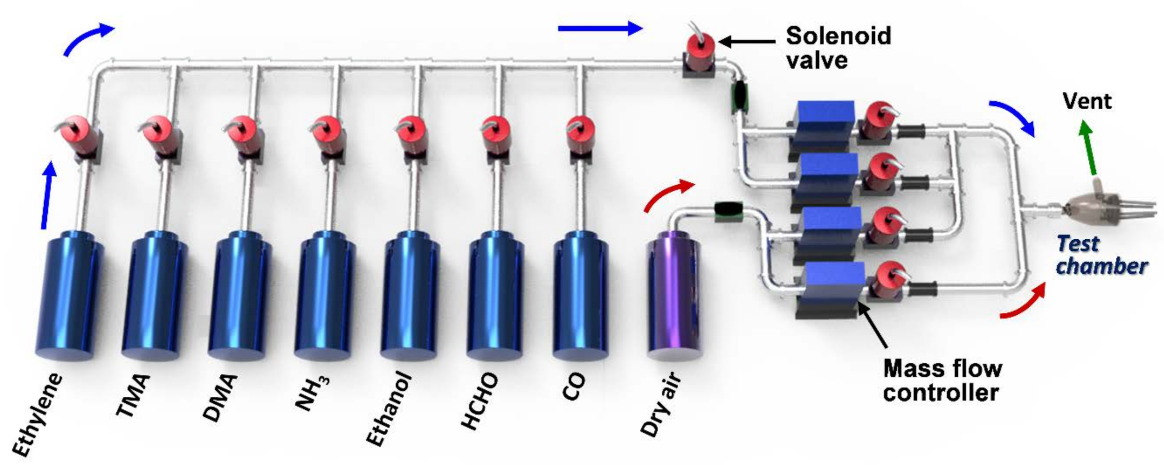

Figure S17. Schematic illustration of the gas sensor measurement system.
